# Quantitative MRI Changes During Weekly Ultra-Hypofractionated Prostate Cancer Radiotherapy With Integrated Boost

**DOI:** 10.3389/fonc.2019.01264

**Published:** 2019-12-04

**Authors:** Marcel A. van Schie, Petra J. van Houdt, Ghazaleh Ghobadi, Floris J. Pos, Iris Walraven, Hans C. J. de Boer, Cornelis A. T. van den Berg, Robert Jan Smeenk, Linda G. W. Kerkmeijer, Uulke A. van der Heide

**Affiliations:** ^1^Department of Radiation Oncology, The Netherlands Cancer Institute, Amsterdam, Netherlands; ^2^Department of Radiation Oncology, University Medical Center Utrecht, Utrecht, Netherlands; ^3^Department of Radiation Oncology, Radboud University Medical Center, Nijmegen, Netherlands

**Keywords:** quantitative MRI, ultra-hypofractionated prostate radiotherapy, MRI changes, T2 mapping, ADC mapping, hormonal therapy

## Abstract

**Purpose:** Quantitative MRI reflects tissue characteristics. As possible changes during radiotherapy may lead to treatment adaptation based on response, we here assessed if such changes during treatment can be detected.

**Methods and Materials:** In the hypoFLAME trial patients received ultra-hypofractionated prostate radiotherapy with an integrated boost to the tumor in 5 weekly fractions. We analyzed T2 and ADC maps of 47 patients that were acquired in MRI exams prior to and during radiotherapy, and performed rigid registrations based on the prostate contour on anatomical T2-weighted images. We analyzed median T2 and ADC values in three regions of interest (ROIs): the central gland (CG), peripheral zone (PZ), and tumor. We analyzed T2 and ADC changes during treatment and compared patients with and without hormonal therapy. We tested changes during treatment for statistical significance with Wilcoxon signed rank tests. Using confidence intervals as recommended from test-retest measurements, we identified persistent T2 and ADC changes during treatment.

**Results:** In the CG, median T2 and ADC values significantly decreased 12 and 8%, respectively, in patients that received hormonal therapy, while in the PZ these values decreased 17 and 18%. In the tumor no statistically significant change was observed. In patients that did not receive hormonal therapy, median ADC values in the tumor increased with 20%, while in the CG and PZ no changes were observed. Persistent T2 changes in the tumor were found in 2 out of 24 patients, while none of the 47 patients had persistent ADC changes.

**Conclusions:** Weekly quantitative MRI could identify statistically significant ADC changes in the tumor in patients without hormonal therapy. On a patient level few persistent T2 changes in the tumor were observed. Long-term follow-up is required to relate the persistent T2 and ADC changes to outcome and evaluate the applicability of quantitative MRI for response based treatment adaptation.

## Introduction

Whole gland dose escalation for prostate cancer has shown to result in increased biochemical control rates, but is associated with increased toxicity (1). Focal dose escalation may benefit patient outcome without compromising toxicity levels compared to conventional treatment. This hypothesis is currently tested in the FLAME trial (2) where patients received an integrated boost up to 95 Gy to the visible tumor in addition to a whole gland dose of 77 Gy in 35 treatment fractions. With advancing insight in prostate tumor radiobiology, hypofractionated prostate radiotherapy is increasingly performed (3, 4). With ultra-hypofractionation, the therapeutic ratio between tumor control and toxicity increases even further due to the low α/β ratio of prostate cancer. Several ultra-hypofractionation trials have demonstrated similar toxicity as compared to standard fractionation, with reduced treatment time (5–8). Also, non-inferiority has already been demonstrated (7, 8). For intermediate to high-risk disease, the combination of ultra-hypofractionation with a focal dose escalation to the tumor as conducted in the FLAME trial may even result in better outcomes. Therefore, ultra-hypofractionation was combined with a focal boost to the tumor to treat intermediate to high-risk prostate cancer in the hypoFLAME trial.

In prostate cancer long term follow-up of at least 5 years is required to evaluate treatment outcome. If changes in the prostate occur at an early stage during treatment and are related to outcome, treatment adaptation for prostate cancer could be considered.

Quantitative MRI is known to reflect tissue characteristics. Diffusion weighted imaging (DWI) and T2 mapping are suitable quantitative MRI techniques to investigate tissue properties in the prostate (9, 10). Through DWI a quantitative apparent diffusion coefficient (ADC) map can be obtained that represents water diffusion between cells and allows to discriminate between malignant and benign prostate tissue. Furthermore, the ADC value of tumor tissue was found to relate to aggressiveness of the disease (11). With T2 mapping a spatial distribution of T2 values can be calculated that are unique to biological tissues. T2 was for example found to correlate with hypoxia (12, 13). Since prostate tumors have different properties from benign prostate tissue, T2 mapping has the potential to discriminate between benign and malignant tissue.

Since quantitative MRI reflects tissue characteristics, tissue changes due to treatment may be visible on quantitative MRI as well. Therefore, quantitative MRI has the potential to generate imaging biomarkers for treatment response assessment. Before investigating this potential role for quantitative MRI, the first step is to identify if any changes in the tumor during treatment can be detected on quantitative MRI.

To identify changes in the prostate during treatment, in the hypoFLAME trial we acquired quantitative MRI data at each weekly fraction of radiation and tracked quantitative MRI values during the course of treatment. Since concurrent hormonal therapy may affect these MRI values (14), we also investigated the influence of hormonal therapy on tissue changes during radiotherapy.

## Methods and Materials

### Patient Characteristics

We collected data of 73 patients from two institutions who participated in the hypoFLAME trial (clinicaltrials.gov NCT02853110). All patients had biopsy-proven, clinically localized, intermediate to high-risk prostate cancer (15). Patients were excluded if they had a contraindication for performing an MRI examination, if no tumor nodule was visible on MRI or if placement of fiducial markers was unsafe. Other exclusion criteria were ≥5 mm seminal vesicle invasion, lymph node or distant metastasis, or an iPSA of more than 30 ng/mL. Also patients that received previous pelvic irradiation or underwent transurethral resection of the prostate (TURP), or patients with an International Prostate Symptom Score (IPSS) > 15 or an World Health Organization (WHO) >2 were not included in the trial. We obtained approval from the institutional review boards and written informed consent from all included patients.

### Treatment Delivery

Patients were treated in the University Medical Center in Utrecht (UMCU, *n* = 36) and the Netherlands Cancer Institute in Amsterdam (NKI, *n* = 37). Dual-arc VMAT treatment was delivered once per week with 35 Gy in five fractions to the prostate, with an integrated focal boost up to 50 Gy to the visible tumor on MRI. Position verification of the prostate was performed prior to each radiation fraction using gold fiducial markers visible on cone-beam CT. In the UMCU 10 out of 36 patients received concurrent hormonal therapy for a period of 6–36 months, in the NKI these were 31 out of 37 patients. Hormonal therapy was typically started 2–6 weeks prior to the start of radiotherapy.

### Scanning Protocol

Prior to treatment patients received a planning CT scan and MRI exam, including a T2-weighted scan and a diffusion weighted imaging (DWI) scan. In the NKI also a T2 mapping sequence was performed. In both institutions patients were scanned on a 3T Philips Ingenia MRI scanner. Specifications of the scanned MRI sequences are listed in Table 1. To track changes in the prostate and tumor during treatment, a weekly repeat MRI exam was scanned at each treatment fraction that included the same image sequences as the pretreatment MRI exam.

**Table 1:** Specifications of MRI sequences in the UMCU and NKI.

	**UMCU**	**NKI**
**T2-WEIGHTED (TSE)**		
Voxel size (mm^3^)		
Acquired	0.6 × 0.7 × 3	0.7 × 0.7 × 3
Reconstructed	0.5 × 0.5 × 3	0.4 × 0.4 × 3
FOV (mm^3^)	200 × 200 × 90 /	282 × 282 × 75–90
	230 × 230 × 141–150	
TE / TR (ms)	90–100 / 3,770–8,620	120 / 3,690–7,930
**T2 MAPPING (MULTI-ECHO SPIN-ECHO)**		
Voxel size (mm^3^)		
Acquired		0.8 × 0.8 × 3 / 1.0 × 1.0 × 3
Reconstructed		0.4 × 0.4 × 3 / 0.6 × 0.6 × 3
FOV (mm^3^)		170 × 170 × 60
TE / TR (ms)		32 / 2,470–4,150
Echo spacing (ms)		16
Echoes (n)		12
**DWI (SINGLE-SHOT EPI)**		
Voxel size (mm^3^)		
Acquired	3.0 × 3.0 × 4	2.3 × 2.4 × 3
Reconstructed	2.5 × 2.5 × 4	1.1 × 1.1 × 3
FOV (mm^3^)	256 × 256 × 66	256 × 256 × 60–66
TE / TR (ms)	62–93 / 3,400–4,940	62 / 2,860–5,410
b-values (s/mm^2^)	0, 100, 300, 500, 800, 1,000	0, 200, 800

*FOV, field of view; TE, echo time; TR, repetition time. For T2 mapping patients were consistently scanned with one of the reported voxel sizes*.

### Calculation of T2 and ADC Maps

The DWI scans were acquired with different protocols as described in Table 1. For consistency between institutions we only considered b-values between 200 and 800 s/mm^2^. In the NKI cohort we calculated the ADC maps using b = 200 and 800 s/mm^2^, in the UMCU cohort we calculated the ADC maps using b = 300, 500, and 800 s/mm^2^.

In the NKI cohort we derived quantitative T2 maps from the T2 mapping sequence. For calculation of the T2 map we applied an in-house developed weighted logarithmic fitting algorithm to determine the T2 value per voxel in the image (16).

### Image Registration

We registered all images to the pretreatment images to allow for tracking of prostate and tumor changes during treatment. All registrations were performed rigidly with in-house developed software using mutual information as the cost function, and registrations were manually adapted whenever required. Within each MRI exam the b = 0 s/mm^2^ image from the DWI was selected, since it contained most anatomical information, and registered to the T2-weighted image. We applied the transformation matrix obtained from registration to the ADC map to register it to the T2-weighted image. From the T2 echo image series the image with echo time closest to the echo time of the T2-weighted image (TE = 120 ms) was selected and registered to the T2-weighted image. We applied the transformation matrix to the T2 map to register it to the T2-weighted image. From each repeat MRI exam we registered the T2-weighted image to the pretreatment T2-weighted image.

### Delineations

We delineated the prostate and the peripheral zone on T2-weighted MRI and labeled the remaining part of the prostate as central gland (CG). The delineation of the tumor was based on multi-parametric MRI. CG, PZ, and tumor together are referred to as ROIs throughout this study.

### Image Analysis

We resampled the registered images to 1 mm isotropic voxels. This allowed for exclusion of an isotropic margin of 2 mm around each ROI that was considered to minimize the impact of residual registration errors. We extracted the median value within each ROI on T2 and ADC. We determined the population median value for each time point during treatment. Per patient we normalized the values to the pretreatment value to examine the relative behavior over time. We stratified by patients with and without hormonal therapy to investigate the influence on T2 and ADC changes during hypofractionated radiotherapy.

On a patient level we identified significant trends using confidence intervals for T2 and ADC defined by literature values. These confidence intervals were derived from test-retest measurements. For T2 we used a confidence interval of 11% as found by van Houdt et al. (17). For ADC we used a value of 47% as recommended by the Quantitative Imaging Biomarkers Alliance (QIBA) (18). These confidence intervals separate real changes in T2 and ADC values from measurement imprecision with 95% confidence. We subsequently determined the number of patients in which T2 and ADC changes were outside the confidence intervals at any time point during treatment and were persistent until week 5.

### Statistics

We performed Wilcoxon signed rank tests to identify if changes per ROI were statistically significant during treatment. We applied a Bonferroni correction to account for multiple testing (nine tests), considering *p* < 0.0056 as significance level. All image analysis and statistical tests were performed using MATLAB (MathWorks, Natick, MA, USA).

## Results

We did not perform analysis on 15 patients for whom <3 out of 6 MRI exams were scanned. Eleven patients were not analyzed since they were scanned with two different DWI scanning protocols during acquisition of pretreatment and repeat MRI. We could not analyze T2 values of four patients since pretreatment T2 maps were not acquired. Table 2 summarizes the number of patients per institution available for analysis.

**Table 2:** Number of patients per institution from which T2 and ADC maps were available for analysis, separated by hormonal therapy (HT or No HT).

	**UMCU**	**NKI**	**All**
**T2**			
HT		21	21
No HT		3	3
All		24	24
**ADC**			
HT	4	24	28
No HT	15	4	19
All	19	28	47

**Figure 1:**
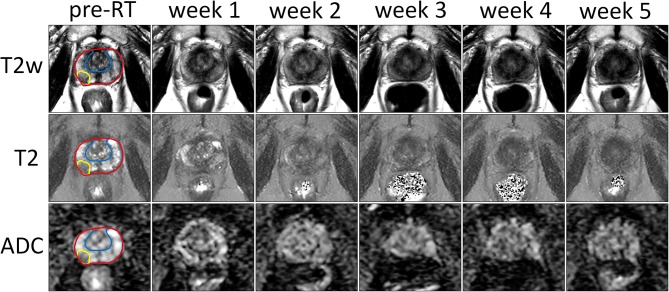
Example of T2-weighted images, and T2 and ADC maps of the prostate prior to treatment (pre-RT) and at each repeat MRI exam (weeks 1–5) of a patient treated at the NKI. The entire prostate, the boundary between PZ and CG and the tumor are delineated in red, blue, and yellow, respectively.

The T2-weighted images, T2 and ADC maps from one patient are shown in Figure 1 for all time points. A decrease in contrast within the prostate can be observed in all three image sequences over the course of treatment, which reduces the conspicuity of the tumor from the surrounding prostate tissue.

Median values of T2 and ADC in the CG, PZ, and tumor during pretreatment imaging are shown in Table 3. We observed statistically significant differences in the CG and PZ between the ADC values in the UMCU cohort and the NKI cohort.

**Table 3:** Population median and interquartile range (between brackets) of median T2 (in ms) and ADC values (in 10^−3^ mm^2^/s) in the CG, PZ, and tumor on pretreatment quantitative MRI.

	**T2 (ms)**	**ADC (10^−3^ mm^2^/s)**	
	**NKI**	**UMCU**	**NKI**
CG	93 (19)	**1.30** (0.13)	**1.09** (0.18)
PZ	110 (26)	**1.37** (0.19)	**1.24** (0.24)
Tumor	80 (9)	1.07 (0.20)	0.90 (0.28)

*Statistically significant differences between institutions are indicated in bold*.

**Figure 2:**
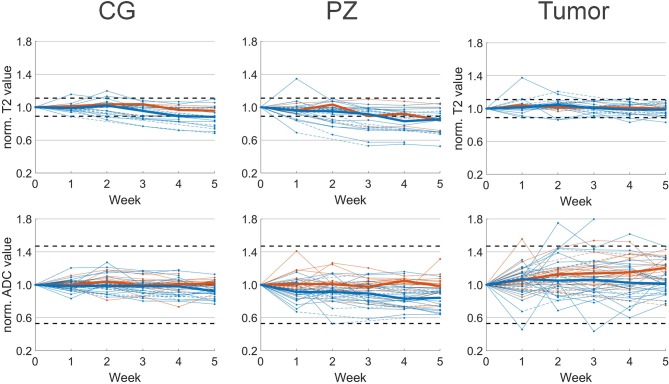
Median normalized T2 (top) and ADC value (bottom) per patient with respect to pretreatment imaging (week 0). Median values of patients with (blue) and without (orange) hormonal therapy are plotted as solid lines. Interpolated values are displayed with dashed lines. Confidence intervals of 11 and 47% for T2 and ADC, respectively, are plotted as horizontal dashed lines.

T2 and ADC values normalized to the pretreatment values are shown in Figure 2. In the CG we observed a median decrease of 12% on T2 and 8% on ADC in patients that received hormonal therapy. T2 and ADC values at week 5 were significantly lower compared to pretreatment values. For patients that received no hormonal therapy, the median ADC value decreased 4% and this was not statistically significant.

In the PZ we observed similar behavior. In patients with hormonal therapy the median T2 and ADC value decreased significantly with 17 and 18%, respectively, while in patients without hormonal therapy we observed a non-significant decrease in ADC of 5%.

In the tumor the behavior was different from CG and PZ. Median increases of 5 and 7% on T2 and ADC maps were found for patients with hormonal therapy, and these were not statistically significant. For patients without hormonal therapy, on ADC we observed a median increase of 20% that was statistically significant.

Due to the low number of patients that were scanned with a T2 mapping sequence and received no hormonal therapy, we did not test statistical significance of T2 changes in these patients. On an individual patient level we found that 14 out of 21 patients who received hormonal therapy, showed persistent T2 changes larger than 11% during treatment. These were 11 patients with persistent changes in the CG, 12 in the PZ and one in the tumor. For the three patients without hormonal therapy, two patients had persistent T2 changes, from which one showed changes in the CG, two in the PZ, and one in the tumor. In total 67% of the 23 patients showed persistent T2 changes during treatment. In contrast, on ADC maps for both patients with and without hormonal therapy we observed no changes outside the confidence interval of 47%.

## Discussion

In this study we analyzed changes in the prostate as observed on quantitative MRI during hypofractionated radiotherapy with an integrated boost to the tumor. Using repeated imaging we observed changes in median T2 and ADC values that depended on the use of hormonal therapy. The changes we observed can explain the reduced tumor conspicuity that is observed after primary radiotherapy. However, depending on hormonal therapy this can be explained by either normalization of tumor characteristics or by a decrease of normal prostate tissue values. For patients who received hormonal therapy, we observed a reduction of T2 and ADC values in the PZ, while values in the tumor did not change significantly. However, for patients who did not receive hormonal therapy, we found that ADC values increased significantly in the tumor, but not in the PZ.

The pretreatment ADC values were significantly different between the two institutions. This may be a consequence of the DWI scanning protocols. The b-values in both protocols were similar with b = 200 and 800 s/mm^2^ in the NKI and b = 300, 500, and 800 s/mm^2^ in the UMCU. However, the acquisition voxel size in the UMCU protocol was 2.2 times larger than in the NKI protocol. This resulted in a different signal to noise ratio and could contribute to differences in ADC values (19).

In the literature a similar variation between ADC values was found. In one study median ADC values in the tumor of 1.08 ± 0.39 · 10^−3^ mm^2^/s (mean ± SD) prior to treatment are reported (20). ADC values in the untreated healthy PZ were 1.8 ± 0.4 · 10^−3^ mm^2^/s. Other studies found values of 1.6 ± 0.2 · 10^−3^ mm^2^/s in the healthy prostate of untreated patients (21, 22). Again differences in DWI protocol as well as image reconstruction methods may have contributed to the existing variation.

We observed different trends in patients that did and did not receive hormonal therapy. Hormonal therapy however correlated with the institution where patients were treated. In the UMCU 4 out of the 19 patients received hormonal therapy, while in the NKI this was 24 out of the 28 patients. Because of this unbalanced distribution we could not separate hormonal therapy from institution to explain the differences in normalized ADC value behavior during treatment. This was also the reason we did not compare the T2 values for patients with and without hormonal therapy in the NKI cohort.

One study describes prostate and tumor changes on MRI during treatment. Foltz et al. (23) reported an early treatment response in the entire prostate and CG, plus a progressive response in the PZ and tumor toward the end of treatment. A statistically significant change in the tumor was found after 6 weeks on ADC. Early treatment response in the tumor was not observed on either T2 or ADC. While there were differences in the overall treatment duration, the frequency of imaging and the time between radiotherapy fractions compared to our study. Our quantitative MRI results indicate similar behavior. We found progressive T2 changes in the PZ and late ADC changes in the tumor. This qualitative comparison is only indicative though, since the use of hormonal therapy was not reported in Foltz et al. (23).

Here we analyzed the T2 and ADC changes in prostate and tumor only during treatment. Dinis Fernandes et al. (16) reported late changes on quantitative MRI in recurrent prostate cancer patients that were scanned at least 2 years after primary treatment. Adjuvant hormonal therapy was given in 82% of the patients but ended at least 1 year before the MRI examination. Changes in CG and PZ regions on both T2 and ADC maps were found and reduced contrast between PZ and tumor on T2 maps was observed. Median T2 values in the CG, PZ and tumor decreased by 29, 19, and 5%, while we observed statistically significant decreased values of 12 and 17% in the CG and PZ and no statistically significant change in the tumor. For ADC values a reduction of 5–9% in CG, PZ and tumor was observed 2 year after treatment. In our study we observed a decrease of 8 and 18% in the CG and PZ in case of hormonal therapy, while an increase of 20% was found in the tumor in absence of hormonal therapy. Based on these findings we expect further reduction of T2 values in the CG and PZ after treatment, as well as post-treatment changes in ADC. Also the treatment fractionation and both timing and duration of hormonal therapy may contribute to the discrepancies between both studies. Follow-up of patients in our study will be required to confirm if changes on T2 and ADC correlate with long term biochemical recurrence free survival.

We implemented a rigid registration method to align all images to the pretreatment T2-weighted image. More accurate registration methods like deformable registration could be more appropriate when registering between MRI exams. Deformable registration would account for possible deformations of the prostate between MRI exams and allow for voxel-level analysis. However, as a result of treatment we experienced intensity changes on T2-weighted images that lead to incorrect deformations we were unable to manually adapt. Therefore, we applied rigid registrations instead and minimized registration inaccuracy via removal of an isotropic margin around each ROI, which required resampling of all images. Since we performed our analysis on ROI level, we expect limited impact of both the registration method and the image resampling on our results.

Using quantitative MRI, on a population level we were able to find significant ADC changes in the intraprostatic tumors of patients that did not receive hormonal therapy during hypofractionated radiotherapy. However, early during treatment, when treatment adaptation could be considered, no significant change was identified in the tumor. We did observe only two individual patients that showed persistent T2 changes in the tumor, while no individual patients showed persistent ADC changes in the tumor. On ADC we did observe several patients with early and progressive trends in the tumor although these trends were within the confidence intervals. If these trends are continued after treatment and exceed the confidence intervals, a possible relation between early treatment response and clinical outcome could be established. Follow-up is therefore desired for assessing the potential role of quantitative MRI for adaptation of hypofractionated radiotherapy based on early treatment response.

## Data Availability Statement

The datasets generated for this study are available on request to the corresponding author.

## Ethics Statement

The studies involving human participants were reviewed and approved by the Medical Ethics Review Committee, University Medical Center, Utrecht, The Netherlands. The patients/participants provided their written informed consent to participate in this study.

## Author Contributions

MS, PH, RS, LK, and UH contributed to the conception and design of the study. PH, FP, HB, CB, LK, and UH contributed to the acquisition of data for the study. MS, PH, GG, IW, and UH contributed to the analysis of data for the study. MS and IW performed statistical analysis. MS wrote the first draft of the manuscript. MS and UH wrote sections of the manuscript. All authors contributed to manuscript revision, read, and approved the submitted version.

### Conflict of Interest

The authors declare that the research was conducted in the absence of any commercial or financial relationships that could be construed as a potential conflict of interest.
